# Intestinal injury and the gut microbiota in patients with *Plasmodium falciparum* malaria

**DOI:** 10.1371/journal.ppat.1011661

**Published:** 2023-10-19

**Authors:** Natthida Sriboonvorakul, Kesinee Chotivanich, Udomsak Silachamroon, Weerapong Phumratanaprapin, John H. Adams, Arjen M. Dondorp, Stije J. Leopold

**Affiliations:** 1 Department of Clinical Tropical Medicine, Faculty of Tropical Medicine, Mahidol University, Bangkok, Thailand; 2 Mahidol-Oxford Tropical Medicine Research Unit, Faculty of Tropical Medicine, Mahidol University, Bangkok, Thailand; 3 Center for Global Health and Infectious Diseases Research, College of Public Health, University of South Florida, Tampa, Florida, United States of America; 4 Centre for Tropical Medicine, Nuffield Department of Clinical Medicine, University of Oxford, Oxford, United Kingdom; 5 Department of Internal Medicine, Division of Infectious Diseases, Amsterdam University Medical Center, location AMC, the Netherlands; Joan and Sanford I Weill Medical College of Cornell University, UNITED STATES

## Abstract

The pathophysiology of severe falciparum malaria involves a complex interaction between the host, parasite, and gut microbes. In this review, we focus on understanding parasite-induced intestinal injury and changes in the human intestinal microbiota composition in patients with *Plasmodium falciparum* malaria. During the blood stage of *P*. *falciparum* infection, infected red blood cells adhere to the vascular endothelium, leading to widespread microcirculatory obstruction in critical tissues, including the splanchnic vasculature. This process may cause intestinal injury and gut leakage. Epidemiological studies indicate higher rates of concurrent bacteraemia in severe malaria cases. Furthermore, severe malaria patients exhibit alterations in the composition and diversity of the intestinal microbiota, although the exact contribution to pathophysiology remains unclear. Mouse studies have demonstrated that the gut microbiota composition can impact susceptibility to *Plasmodium* infections. In patients with severe malaria, the microbiota shows an enrichment of pathobionts, including pathogens that are known to cause concomitant bloodstream infections. Microbial metabolites have also been detected in the plasma of severe malaria patients, potentially contributing to metabolic acidosis and other clinical complications. However, establishing causal relationships requires intervention studies targeting the gut microbiota.

## Introduction

Malaria remains a significant global health burden, with an estimated 247 million cases and 620.000 deaths in 2021 [1]. The majority of severe and fatal malaria cases are caused by *Plasmodium falciparum*, one of the 5 malaria parasite species affecting humans. A crucial aspect of the pathophysiology of severe falciparum malaria is the extensive sequestration of parasitized red blood cells in the microcirculation, which impairs microcirculatory blood flow and leads to dysfunction of vital organs [2]. Additionally, endothelial activation and glycocalyx dysfunction are believed to further compromise tissue perfusion [3]. The degree of microvascular dysfunction can be assessed by directly observing the microcirculation and estimating the sequestered parasite biomass using plasma *P*. *falciparum* histidine-rich protein 2 (*Pf*HRP2) [4,5]. Notably, microvascular sequestration of parasitized red blood cells is prominent in the gut [4,6], affecting gut perfusion and potentially disrupting tight and adherent junctions in the gut epithelium. This disruption compromises the gut’s barrier function and facilitates the translocation of enteric bacteria into the bloodstream, as previously reviewed [7].

The microbiome consists of a diverse group of bacteria, archaea, fungi, protozoa, and viruses. Among the human-associated microbial communities, the gut microbiota is the largest and most heterogeneous [8]. The gut microbiota has been implicated in numerous physiological processes, including energy homeostasis, metabolism, gut epithelial health, immunologic activity, and neurobehavioral development [9]. Furthermore, the microbiota’s composition may play a role in the pathogenesis of various infectious diseases, such as influenza [10], bacterial sepsis [11], *Clostridium difficile* enteritis [12], and in malaria as shown in prior reviews of mouse studies [13,14]. Previous mouse studies have demonstrated that different gut microbiota compositions result in varying disease severities [15–18], in particular, in mouse models of malaria [15]. Several studies in both human and rodent malaria have shown changes in gut microbiota composition during *Plasmodium* infections, with an association observed with disease severity. The causal relationship between gut microbiota composition and disease severity in humans remains unclear, but it may offer new insights into the pathophysiology of severe malaria and potential targets for intervention.

In this review, we focus on the evidence from clinical studies in patients with malaria for intestinal injury, changes in microbiota composition, and their potential consequences in *P*. *falciparum* malaria. These consequences include the translocation of bacteria and their metabolites across the gut barrier and alterations in gut immunological functions. Furthermore, we briefly discuss potential interventions targeting the gut microbiota.

In a parallel review, Mandal and Schmidt discuss the evidence and insights from laboratory and experimental studies into the interaction between the host gut microbiome and malaria.

## Parasite-induced intestinal injury

Patients with malaria commonly experience gastrointestinal symptoms such as nausea, vomiting, abdominal pain, and diarrhea. Intestinal damage occurs through a complex pathological cascade, primarily driven by the extensive sequestration of parasitized red blood cells in the splanchnic microcirculation of severe malaria patients. For a summary of the mechanisms involved, see Table 1.

**Table 1:** Evidence from clinical studies in patients with malaria for a pathological cascade of parasite-induced intestinal injury, impaired intestinal barrier function, translocation of bacteria and metabolites into the bloodstream, and an altered gut microbiota composition.

Study	Population	Organ/tissue	Measurement	Finding	Ref.
**Parasite-induced intestinal injury**					
**Pongponratn and colleagues (1991)**	Adults with fatal malaria (*n* = 39)	Brains, hearts, lungs, small intestines	Autopsy	Sequestration of parasitized red blood cells in mucosal villi capillaries in the intestines	[6]
**Milner and colleagues (2015)**	Children with fatal cerebral malaria (*n* = 103)	Brain, intestines, and others	Autopsy	Extensive sequestration parasitized red blood cells in the capillary network of the lamina propria in the intestines	[33]
**Dondorp and colleagues (2008)**	Adults with severe malaria (*n* = 43)	Rectal microcirculation	In vivo video-microscopy	Obstruction of the intestinal microcirculation related to disease severity	[4]
**Hanson and colleagues (2015)**	Adults with severe malaria (*n* = 142)	Rectal microcirculation	In vivo video-microscopy	Obstruction of the intestinal microcirculation	[34]
**Impaired intestinal barrier function**					
**Molyneux and colleagues (1989)**	Adults with uncomplicated malaria (*n* = 12)	Gastric and small intestine permeability	Sugar absorption tests	Severe, yet reversible impairment of mucosal barrier function in malaria	[35]
**Wilairatana and colleagues (1997)**	Adults with severe malaria (*n* = 7), uncomplicated malaria (*n* = 14), healthy controls (*n* = 11)	Gastric and small intestine permeability	Sugar absorption tests	Gastrointestinal permeability in patients with severe and uncomplicated malaria, returning to normal on recovery	[36]
**Olsson and colleagues (1969)**	Soldiers with severe malaria (*n* = 20)	Intestines	Small-bowel biopsy and sugar absorption tests	Vascular congestion and oedema of the lamina propria and impaired absorption of sugar probes	[37]
**Olupot-Olupot and colleagues (2013)**	Children with severe malaria (*n* = 257)	Circulating markers	Endotoxins/I-FABP	Endotoxaemia observed in 71 (27.6%) children	[32]
**Sarangam and colleagues (2022)**	Children with severe malaria (*n* = 598) and healthy controls (*n* = 120)	Circulating markers	TFF3, I-FABP	Intestinal injury biomarkers significantly elevated in children with severe malaria associated with mortality	[26]
**Translocation of intestinal bacteria into the bloodstream**					
**Berkley and colleagues (2009)**	Children with severe malaria (*n* = 3,068), healthy controls (*n* = 592)	Whole blood	Blood cultures	Invasive bacterial infection detected in 127 (6%) of 2,048 consecutive parasitaemic admitted children (95% CI, 5.2%–7.3%)	[38]
**Bassat and colleagues (2009)**	Children with malaria (*n* = 7,043)	Whole blood	Blood cultures	Children with malaria with bacteraemia (5.4% of cases) on admission	[39]
**Nyen and colleagues (2016)**	Adults with malaria (*n* = 67)	Whole blood	Blood cultures	Adults with malaria with bacteraemia (13% of cases) on admission	[29]
**Aung and colleagues (2018)**	Adults with malaria (*n* = 87)	Whole blood	Blood cultures	Adults with severe malaria with clinically significant bacteraemia (15% of cases) on admission	[40]
**Phu and colleagues (2020)**	Adults with severe malaria (between 1991 and 2003) (*n* = 845)	Whole blood	Blood cultures	Adults with severe malaria with bacteraemia (1.0% of cases) on admission	[27]
**Leopold and colleagues (2019)**	Adults with severe malaria (*n* = 60), uncomplicated malaria (*n* = 47), healthy controls (*n* = 45)	Plasma	LC-MS	Microbial metabolites detected in the plasma of patients with uncomplicated and severe malaria	[31]
**Altered gut microbiota**					
**Mandal and colleagues (2021)**	Children with severe malaria (*n* = 40), healthy controls (*n* = 35)	Stool samples	16S ribosomal RNA	Changes in the gut microbiota related to severity of disease	[18]
**Leopold and colleagues (2021) (preprint)**	Adults with severe malaria (*n* = 29), uncomplicated malaria (*n* = 23), local healthy controls (*n* = 34)	Stool samples	16S ribosomal RNA	Changes in the gut microbiota related to severity of disease	[25]

TFF3, trefoil factor 3; I-FABP, intestinal fatty acid binding protein; LC-MS, liquid-chromatography mass spectrometry.

The splanchnic circulation supplies blood to abdominal organs, including the liver, spleen, stomach, pancreas, small intestine, and large intestine. It is perfused by 3 branches of the abdominal aorta: the coeliac artery, superior mesenteric artery, and inferior mesenteric artery.

The splanchnic blood flow is primarily influenced by systemic vascular resistance and cardiac output. Under normal conditions, splanchnic blood flow accounts for about 25% to 30% of cardiac output, but it can vary depending on factors such as recent feeding or physiological stress [19].

Hemodynamic shock is rare in severe malaria, as both systemic vascular resistance and cardiac output are typically maintained at adequate levels despite infections with a large parasite load. This is different from the macrovascular changes seen in bacterial sepsis. The key feature of septic shock is significant peripheral arteriolar vasodilation. This leads to low systemic vascular resistance, high cardiac output, severe hypotension, and shock, with subsequent inadequate tissue perfusion. Studies have shown that systemic vascular resistance remains at adequate levels in both uncomplicated and severe malaria, possibly due to the vasoconstrictive effects of cell-free hemoglobin released during red cell hemolysis [20]. Mildly elevated systemic vascular resistance has been observed in fatal cases of severe falciparum malaria [21]. It is unlikely that macrovascular changes contribute to splanchnic bed hypoperfusion and intestinal injury in severe malaria.

Sequestration of parasitized red blood cells, however, significantly affects the blood flow in the intestines’ microcirculation. Autopsy studies have shown that, given the total blood volume of the splanchnic circulation, the intestines represent a significant proportion of the total body sequestration [6,22]. Video-microscopy of the rectal circulation in living patients with severe malaria has confirmed widespread microcirculatory obstruction in the intestines [4]. Microvascular sequestration of parasitized red blood cells is believed to cause intestinal damage and contribute to the development of hyperlactatemia in severe malaria.

Additional mechanisms that may contribute to intestinal injury include ischemia-reperfusion injury, intestinal inflammation, and mast cell activation. Mast cell activation can damage the gut barrier, both physically and immunologically, through the release of Th2 cytokines that affect the defence against bacteria that may translocate from the intestine [23,24]. This may lead to disruption of the tight and adherent junctions between gut epithelial cells, further compromising gut barrier function, as previously reviewed [7].

## Impaired intestinal barrier function

Intestinal injury in patients with malaria can progress to impaired intestinal barrier function, as evidenced by increased intestinal permeability and abnormal markers of intestinal integrity, Table 1. In adults with severe malaria, observational studies have shown that patients with a high parasite biomass exhibit reduced enterocyte integrity, indicated by decreased plasma L-citrulline, a marker produced by enterocytes in the small intestine [25]. In pediatric patients with malaria, elevated levels of trefoil factor 3 (TFF-3) and intestinal fatty acid binding protein (I-FABP), markers of intestinal injury, are associated with severe malaria and an increased risk of death [26].

Impairment of the gut barrier can increase the likelihood of translocation of enteric bacteria into the bloodstream, leading to concomitant bacteraemia and sepsis. In children with severe malaria in Africa, concurrent invasive bacterial infections are described, involving bacteria such as *Streptococcus pneumoniae*, nontyphoidal *Salmonella*, and *Escherichia coli* [27–30]. Additionally, studies have observed elevated plasma concentrations of bacterial metabolites in patients with severe falciparum malaria [31,32].

## Gut microbiota alterations in malaria

The composition of the gut microbiota can potentially influence the progression of malaria infection through various mechanisms. One important mechanism is colonization resistance, which refers to the ability of the gut microbiota to prevent the overgrowth of harmful bacteria by employing different mechanisms, including the reduction of gut pH [41–43]. Perturbations in the gut microbiota can disrupt this balance and lead to the overgrowth and translocation of harmful bacteria, resulting in the dissemination of their metabolites into the bloodstream. This phenomenon has been observed in the development of *C*. *difficile* enteritis following broad-spectrum antibiotic treatment [44]. The gut microbiota also plays a role in modulating the immune response, both innate and adaptive, and changes in the microbiota due to antibiotic treatment have been shown to weaken the immune response to certain pathogens [45]. Additionally, the gut microbiota contributes to gut barrier function through the production of short-chain fatty acids [11].

In an observational study conducted in Bangladesh, the gut microbiota composition of adult patients with severe and uncomplicated falciparum malaria was compared to healthy volunteers [25]. The study utilized sequencing of the V4 region of the 16S rRNA gene amplified from fecal DNA [25]. Patients with severe malaria showed a significant enrichment of potentially pathogenic *Enterococcus* and *Escherichia/Shigella* species in their gut microbiota, pathogens that are known to be able to cause bloodstream infections [25]. Furthermore, an abundance of lactate-producing species, including *Bacteroides*, *Streptococcus* spp., and *Lactobacillus* spp., in the gut microbiota was associated with the severity of metabolic acidosis, which is a strong predictor of fatal outcomes in severe malaria [25,31]. However, the causal mechanisms underlying these associations remain unclear, and it is important to consider various environmental and patient-related factors that can influence the composition of the gut microbiota, such as age, diet, comorbidities, and prior treatment with antibiotics or antimalarials. To date, no intervention studies targeting the gut microbiota in patients with malaria have been conducted.

Furthermore, mouse models have shown changes in the gut microbiota composition following infection with different *Plasmodium* species. In Swiss Webster and C57BL/6 (B6) mice infected with *Plasmodium yoelii*, a reduction in the Firmicutes/Bacteroidetes ratio and a decrease in Proteobacteria were observed [46]. Another study in B6 and BALB/c mice infected with *Plasmodium berghei* revealed a decrease in Firmicutes and, specifically in one mouse strain, an increase in Proteobacteria and Verrucomicrobia [47]. In C57BL/6 mice, liver damage and bile acid depletion correlated with an increase in gut bacterial diversity during and after infection with *P*. *yoelii*, suggesting a potential role of bile acids in shaping the gut microbiota [48]. These findings highlight the alterations of the gut microbiota in malaria infection.

## Impact of gut microbiota on *Plasmodium* infections

The role of the gut microbiota in influencing the immune response to malaria has been studied primarily in mouse models, with some evidence suggesting a similar effect in human malaria. Here, we summarize important findings from recent experimental studies, but for more insights into the interaction between the host gut microbiome and malaria, see the parallel review by Mandal and Schmidt, where the experimental evidence is further elaborated.

Mouse models of *Plasmodium berghei* or *Plasmodium yoelii* infection, including BALB/c and C57BL/6 mice, have been used to investigate the relationship between the gut microbiota and malaria pathogenesis. Studies have shown that mice colonized with the gut pathobiont *Escherichia coli* O86:B7, which expresses α-galactosyl, produce antibodies (against α-galactosyl) that cross-react with *Plasmodium* sporozoites, and could mediate clinical protection against malaria infection [49].

The composition of the gut microbiota in mouse models appears to impact parasite burden and fatality rates following infection with various *Plasmodium* species. In one study, fecal content from mice with different susceptibility to *P*. *yoelii* infection was transplanted into germ-free mice, demonstrating that resistance to infection could be transferred through fecal transplant [15]. In this study, relative protection against *Plasmodium* correlated with the abundance of *Lactobacillus* and *Bifidobacterium* bacteria [15].

Differences in parasite burden and bacterial community composition have been observed between different strains of mice. For example, Taconic mice showed lower peak parasite burden and faster recovery compared to Charles River mice, suggesting that the host–microbiota interaction plays a role in parasite burden rather than genetics or environmental factors [50]. The study also identified differences in gene expression, including the cell surface receptor basigin, which may link the gut microbiome and malaria resistance.

Intestinal helminth infections, such as hookworm (*Ancylostoma duodenale*, *Necator americanus*), ascaris (*Ascaris lumbricoides*), and whipworm (*Trichuris trichiura*), have also been associated with malaria susceptibility [51], although the data are not conclusive.

Studies on hookworm coinfections largely indicate increased susceptibility to malaria. A study from Ethiopia reported that the intensity of hookworm (and trichuriasis) coinfections was associated with increased densities of both *P*. *falciparum* and *P*. *vivax* [51]. Other studies from Uganda and Zimbabwe investigating hookworm coinfection reported early *P*. *falciparum* parasitemia [52,53], while another study from Uganda showed no association between hookworm infection and early or delayed parasitemia [54]. Most studies on *Ascaris lumbricoides* coinfections suggest a protective effect against malaria. A negative correlation was observed between the intensity of *A*. *lumbricoides* infection and *P*. *falciparum* and *P*. *vivax* parasitemia [51]. Another study from Thailand suggested protection from coinfection with *Ascaris lumbricoides* against the development of cerebral malaria or renal failure in patients with severe malaria [55,56]. However, a study from Cameroon reported no association between intestinal helminths (including ascaris and hookworm) and the clinical outcome of malaria [57].

Proposed mechanisms for the protective effect of ascaris infection include endothelial cell receptor down-regulation and the production of IgE-anti-IgE immune complexes that reduce the severity of falciparum malaria. Endothelial cell receptor down-regulation reduces parasite erythrocyte cytoadherence or selective splenic parasite clearance, thus reducing the proportion of virulent *P*. *falciparum* strains. It has also been suggested that IgE-anti-IgE immune complexes resulting from helminth infections reduce the severity of falciparum malaria and can mediate tolerance to the malaria parasite through the CD23/NO pathway [55]. Studies examining malaria in pregnancy have shown a negative correlation between *A*. *lumbricoides* infection and the risk of *P*. *vivax* malaria [58], while hookworm has been associated with an increased incidence of *P*. *falciparum* but not *P*. *vivax* parasitemia [56,59]. Overall, based on these epidemiological observations the relationship between intestinal helminth infections and malaria susceptibility or severity remains inconclusive.

## Potential interventions targeting the gut microbiota

Therapies aimed at modifying the gut microbiota composition could be potential interventions to influence susceptibility to and severity of *Plasmodium* infections. These interventions include probiotics, selective digestive tract decontamination, fecal transplants, and antibiotics.

### Probiotics

Probiotics are live microorganisms that can modify the gut microbiota. When combined with prebiotics, which support their growth, they are known as synbiotics [60,61]. Common probiotic bacteria include *Lactobacilli* spp., *Bifidobacteria* spp., *Saccharomyces boulardii*, and *Bacillus coagulans*. Probiotics and synbiotics have been suggested to reduce pathobionts through colonization resistance, prevent bacterial translocation, degrade toxins, and modulate the immune response [60].

While probiotics and synbiotics have been primarily studied for their potential in sepsis, their effects on *Plasmodium* infections remain largely unexplored in human studies. However, experimental mouse studies have shown promising results and have suggested a positive effect on time to death, reduction of bacteraemia, and improved gut wall integrity [62]. A large Indian study demonstrated a protective effect of a synbiotic containing *Lactobacillus plantarum* plus fructooligosaccharide on the prevalence of sepsis in neonates and infants. Although an earlier meta-analysis suggested a beneficial effect of probiotics on the prevention of ventilator-associated pneumonia (VAP) [63], a recent large multicenter, double-blinded, randomized controlled trial comparing the efficacy of the probiotic *Lactobacillus rhamnosus* GG (LGG) versus placebo in preventing VAP did not confirm these findings [64].

There are no human studies on the use of probiotics in patients with malaria.

Probiotics containing *Lactobacillus* and *Bifidobacterium* have demonstrated a beneficial effect in reducing *P*. *yoelii* parasitemia in another experimental mouse study [15]. The administration of *Lactobacillus casei* reduced the severity of *Plasmodium chabaudi* infection [65]. In a mouse model of *Plasmodium berghei*, the efficacy of *Lactobacillus casei* as adjuvant therapy to chloroquine, an antimalarial drug, was evaluated and showed a reduction in peripheral blood parasitemia with probiotic treatment [66].

### Selective digestive tract decontamination

Selective digestive tract decontamination (SDD) involves the use of non-absorbable antimicrobials applied daily in the oropharynx and gastrointestinal tract [11]. SDD has been shown to reduce nosocomial infections and lower mortality in large trials involving critically ill patients in intensive care units in the Netherlands. It is now a standard infection prevention measure in Dutch ICUs [67]. SDD prevents colonization of potentially pathogenic microorganisms, including gram-negative aerobic microorganisms and *Staphylococcus aureus*, in the oropharynx and intestines [68]. SDD has not been studied as an adjunctive therapy in malaria and has also not been investigated in animal models of malaria.

### Other interventions: Fecal transplants and antibiotics

Fecal microbiota transplantation (FMT) involves the administration of a solution of fecal material from a healthy donor into the intestinal tract of a recipient through a feeding tube to restore the gut microbiota [69]. FMT is currently used in the treatment of severe *C*. *difficile* infections (CDI) [70]. It has also been studied in patients with other causes of diarrhea or sepsis [71]. However, FMT has not been investigated in human or animal models of malaria.

Antibiotics represent a potential intervention for the treatment of bacterial coinfections in severe malaria. World Health Organization management guidelines recommend empirical broad-spectrum antibacterial therapy for all children diagnosed with severe falciparum malaria in malaria-endemic areas [72].

## Conclusions

Intestinal injury and the gut microbiota appear to play a role in the severity and outcome of falciparum malaria. Mouse studies show that altering the microbiota affects susceptibility to *Plasmodium* infections. In humans, malaria leads to changes in the gut microbiota, including an increase in pathogens associated with severe disease and bacterial infections. Studies in patients with malaria show parasite-induced intestinal injury. Subsequently, impaired intestinal barrier function could allow translocation of gut microbiota and microbial metabolites into the bloodstream, potentially leading to concomitant sepsis. Observational studies in humans provide associations between the microbiota and disease severity or protection. Possible interventions include probiotic therapy, selective digestive tract decontamination, or fecal transplantation therapy. However, intervention studies are needed to establish causal relationships.
